# Targeting Common Inflammatory Mediators in Experimental Severe Asthma and Acute Lung Injury

**DOI:** 10.3390/ph17030338

**Published:** 2024-03-05

**Authors:** Andrei Gheorghe Vicovan, Diana Cezarina Petrescu, Aurelia Cretu, Cristina Mihaela Ghiciuc, Daniela Constantinescu, Elena Iftimi, Georgiana Strugariu, Codrina Mihaela Ancuta, Cezar-Cătălin Caratașu, Carmen Solcan, Celina Silvia Stafie

**Affiliations:** 1 Department of Morpho-Functional Sciences II—Pharmacology and Clinical Pharmacology, Faculty of Medicine, Grigore T. Popa University of Medicine and Pharmacy of Iasi, 16 Universitatii Street, 700115 Iasi, Romania; andrei-gheorghe.vicovan@umfiasi.ro (A.G.V.); diana.petrescu@umfiasi.ro (D.C.P.); aurelia.cretu@umfiasi.ro (A.C.); 2 “Saint Mary” Emergency Children Hospital, 700887 Iasi, Romania; 3 Department of Immunology, Faculty of Medicine, Grigore T. Popa University of Medicine and Pharmacy, 700115 Iasi, Romania; d.constantinescu@umfiasi.ro (D.C.); elena_iftimi@d.umfiasi.ro (E.I.); 4 2nd Rheumatology Department, Clinical Rehabilitation Hospital, 700664 Iasi, Romania; georgiana_strugariu@yahoo.com (G.S.); codrina_ancuta@yahoo.com (C.M.A.); 5 Rheumatology Department, University of Medicine and Pharmacy “Grigore T. Popa”, 700115 Iasi, Romania; 6 Advanced Research and Development Center for Experimental Medicine (CEMEX), Grigore T. Popa University of Medicine and Pharmacy of Iasi, 16 Universității Street, 700115 Iasi, Romania; caratasu.catalin@umfiasi.ro; 7 Department IX—Discipline of Histology, Embryology and Molecular Biology, Faculty of Veterinary Medicine, “Ion Ionescu de la Brad” University of Life Sciences, 700490 Iasi, Romania; carmensolcan@yahoo.com; 8 Department of Preventive Medicine and Interdisciplinarity—Family Medicine Discipline, Faculty of Medicine, Grigore T. Popa University of Medicine and Pharmacy of Iasi, 16 Universitatii Street, 700115 Iasi, Romania; celina.stafie@umfiasi.ro

**Keywords:** Secukinumab, asthma, acute lung injury, Lipopolysaccharide, Interleukin 17, mice

## Abstract

Neutrophils, known to be mobilized and activated in high amounts through Il-17 stimulation, are a key factor for clinical manifestation and imbalance of redox systems favoring a dominant oxidative state in both severe asthma and acute lung injury (f). The aim of this study was to evaluate in mice, the effect of Secukinumab (SECU) in a model of ovalbumin-induced asthma exacerbated with LPS administration to induce ALI, compared to dexamethasone (DEXA), already known for its benefit in both asthma and ALI. Results on cytokine levels for specific Th1, Th2 and Th17 revealed an interplay of immune responses. For Th1 effector cytokines in BALF, DEXA treatment increased TNF-α levels, but TNF-α was not modified by SECU; DEXA and SECU significantly decreased IFN-γ and IL-6 levels. For typical Th2 cytokines, DEXA significantly increased Il-4, Il-5 and Il-13 levels, while SECU significantly inhibited Il-5 levels. Both SECU and DEXA significantly decreased Il-17 levels. Cytokine level changes in lung tissue homogenate were partly similar to BALF cytokines. Conclusion: in addition to DEXA, SECU possesses the ability to modulate inflammatory cytokine release and to decrease Th17 responses in ALI overlapped on exacerbated asthma in mice.

## 1. Introduction

At first sight, severe asthma and acute lung injury (ALI) do not seem to have much in common besides the clinical manifestation of acute respiratory failure. However, the inhibition of IL-17 has been demonstrated to ameliorate de Lipopolysaccharide (LPS) exacerbated asthma in murine models [1,2,3,4] and, at the same time, to exert protective effects on LPS-induced ALI in mice [5]. Moreover, asthma is associated with an increased risk of pneumonia [6], a predisposing clinical factor for ALI [7]. Also, an impaired oxidative status was established in both exacerbated asthma and ALI. On the one hand, the imbalance of redox systems favoring a dominant oxidative state in asthma is determinant for the pathophysiology of airflow limitation, hyper-reactivity and airway remodeling, all of them factors of disease severity [8,9]. On the other hand, the antioxidant state is severely depressed in patients with ALI at the onset and during the course of the disease [10] with a redox imbalance favoring ROS production [11]. A key factor for this impairment in both pathologies are the neutrophils [12,13] known to be mobilized and activated in high amounts through Il-17 stimulation [14].

The pharmacological rationale for targeting IL-17 in certain asthma phenotypes is legitimated by its involvement in the development of severity characteristics: it reduces the sensitivity to corticosteroids [15], stimulates expression of the mucin MUC5B (a marker for mucin overproduction) [16] and drives hyperresponsiveness [17]. Il-17 plays a key role in the cytokine storm that occurs in ALI of any etiology, it participates in the development of ALI [18] and regulates lung inflammation in LPS-induced ALI mice from a mechanistically point of view [19]. Il-17 level is associated with alveolar inflammation and a poor prognosis [20] and it is increased in severe cases.

Previous experimental research revealed beneficial effects of anti-Il-17 in LPS exacerbated asthma in murine models [1,2,3,4] and an alleviation of ALI inflammation [21], possibly by reducing the expression of cytokines and oxidative stress [5]. Consequently, IL-17 can be considered a legitimate target in the management of acute respiratory failure associated either with severe asthma or with ALI.

Secukinumab (SECU) is a promising fully human anti-IL-17 monoclonal antibody that selectively neutralizes IL-17A, approved for the treatment of moderate to severe plaque psoriasis, psoriatic arthritis and ankylosing spondylitis. SECU exhibited also modulatory immune effects in different rodent experimental models [22,23,24,25,26]. Moreover, high-dose SECU showed protective effects even against severe sepsis induced ALI on rat models by inhibiting the activation of the IKBα/NFκB inflammatory pathway [27]. However, contrary to the previous mentioned experimental results, we found data from a pharmaceutical company noting that Secukinumab is able to neutralize cynomolgus, rhesus and marmoset monkey IL-17A, but not the rodent IL-17A (Secukinumab—report on deliberation results, https://www.pmda.go.jp/files/000216876.pdf, accessed on 20 January 2024).

In the context of conflicting data, there is a gap in the research regarding the effects of SECU on LPS-induced ALI or LPS exacerbated asthma in murine models. Considering the favorable SECU safety profile in clinical trials [28], the aim of our study was to evaluate the potential of SECU in a particular model of allergic asthma in mice, that through LPS exacerbation evolves to pneumonia-like inflammatory status and, finally develops ALI. This model has the main elements of the clinical evolution of severe asthma. A positive control treatment was preferred, dexamethasone (DEXA), already known for its beneficial effects in both asthma and ALI [29,30].

## 2. Results

### 2.1. Effects of SECU on the Level of Ovalbumin (OVA)-Specific IgE in Serum

**Figure 1:**
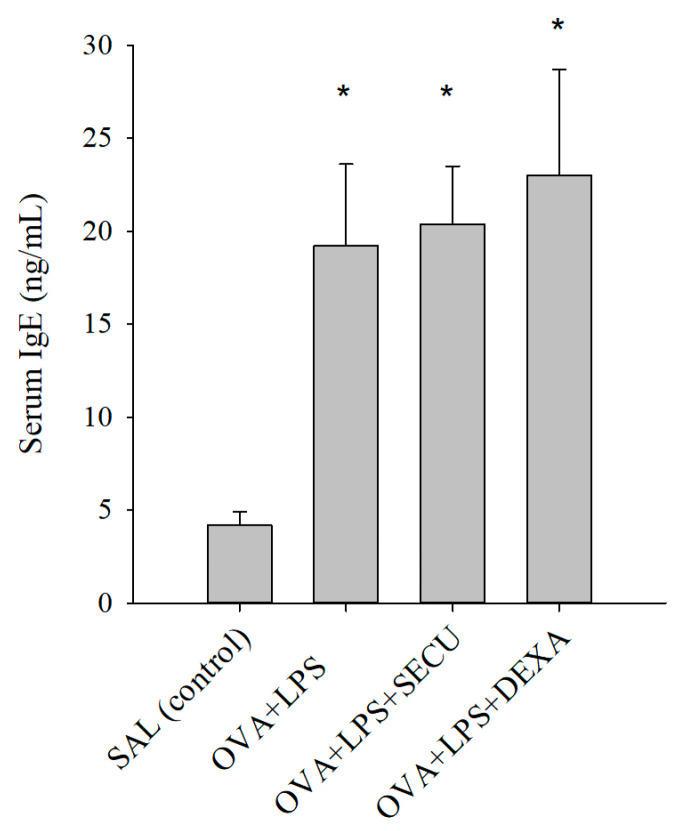
IgE concentration in serum (ng/mL). Values are expressed as mean ± standard error; *n* = 6. * *p* < 0.05 vs. control as determined by one-way ANOVA, followed by the Holm–Sidak method for multiple comparisons. SAL: normal control group; OVA + LPS: positive control for disease; OVA + LPS + SECU: Secukinumab treated group; OVA + LPS + DEXA: Dexamethasone treated group.

In Figure 1, the increased level in OVA-specific IgE in the serum of OVA + LPS (positive control for disease), OVA + LPS + SECU (Secukinumab treated group) and OVA + LPS + DEXA (Dexamethasone treated group) in contrast with very low level in the SAL (normal control) group is depicted, which confirmed the existence of OVA sensitization in the OVA exposed groups. There were no statistically significant modifications induced by the treatment with SECU or DEXA, compared with the positive control in mice with asthma and acute lung injuries.

### 2.2. Effects of Secukinumab on Bronchoalveolar Lavage Fluid (BALF)

#### 2.2.1. Cell Count in BALF

**Figure 2:**
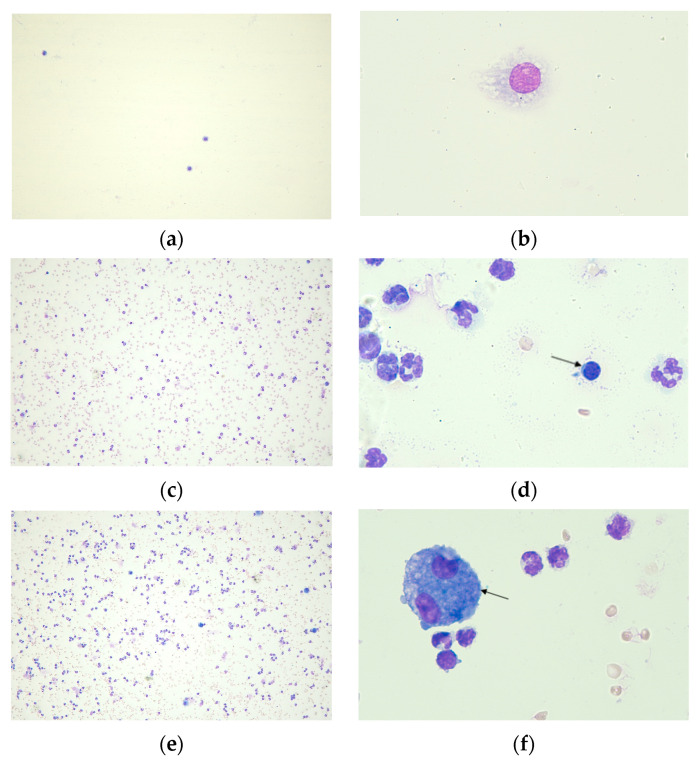

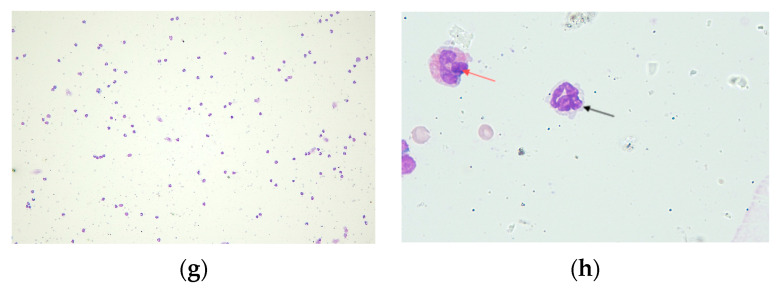
Differential cell count: (**a**) SAL group: alveolar macrophages, optical microscopy, ×10 dry objective; (**b**) SAL group: alveolar macrophage, optical microscopy, ×100 immersion objective; (**c**) OVA + LPS group: cellularity, optical microscopy, ×10 dry objective; (**d**) OVA + LPS group: neutrophils and a lymphocyte (arrow), optical microscopy, ×100 immersion objective; (**e**) OVA + LPS + SECU group: cellularity, optical microscopy, ×10 dry objective; (**f**) OVA + LPS + SECU group: binucleated alveolar macrophage (arrow) and neutrophils, optical microscopy, ×100 immersion objective; (**g**) OVA + LPS + DEXA group: cellularity, optical microscopy, ×10 dry objective; (**h**) OVA + LPS + DEXA group: one neutrophil (black arrow) and one eosinophil (red arrow), optical microscopy, ×100 immersion objective. SAL: normal control group; OVA + LPS: positive control for disease; OVA + LPS + SECU: Secukinumab treated group; OVA + LPS + DEXA: Dexamethasone treated group.

Figure 2 shows the differences in inflammatory cells in BALF for macrophages, neutrophils, lymphocytes and eosinophils.

**Figure 3:**
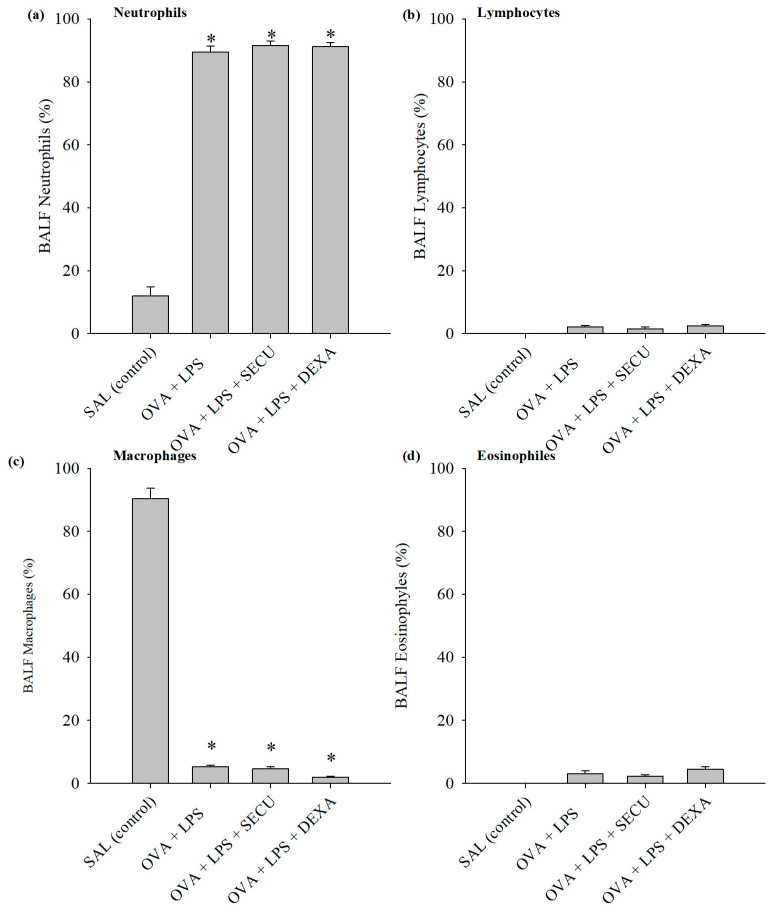
Description of the differential cell counts in percentage for (**a**) macrophages, (**b**) neutrophils, (**c**) lymphocytes and (**d**) eosinophils. Values are represented as mean ± standard error; *n* = 6. One-way ANOVA: * *p* < 0.05 vs. SAL group; followed by the Holm–Sidak method for multiple comparisons. SAL: normal control group; OVA + LPS: positive control for disease; OVA + LPS + SECU: Secukinumab treated group; OVA + LPS + DEXA: Dexamethasone treated group.

The evaluation of the differential cell counts for macrophages, neutrophils and eosinophils revealed a percentual increase in neutrophils in the OVA + LPS, OVA + LPS + SECU and OVA + LPS + DEXA groups compared to the SAL group (*p* < 0.05) (Figure 3).

There were no significant differences among the counts of lymphocytes and eosinophils in any of the groups. Also, the macrophages from OVA + LPS and OVA + LPS + SECU groups were not significantly different compared to the SAL group. Only the OVA + LPS + DEXA group showed a decrease compared to all the other three groups.

#### 2.2.2. Cytokines in BALF

**Figure 4:**
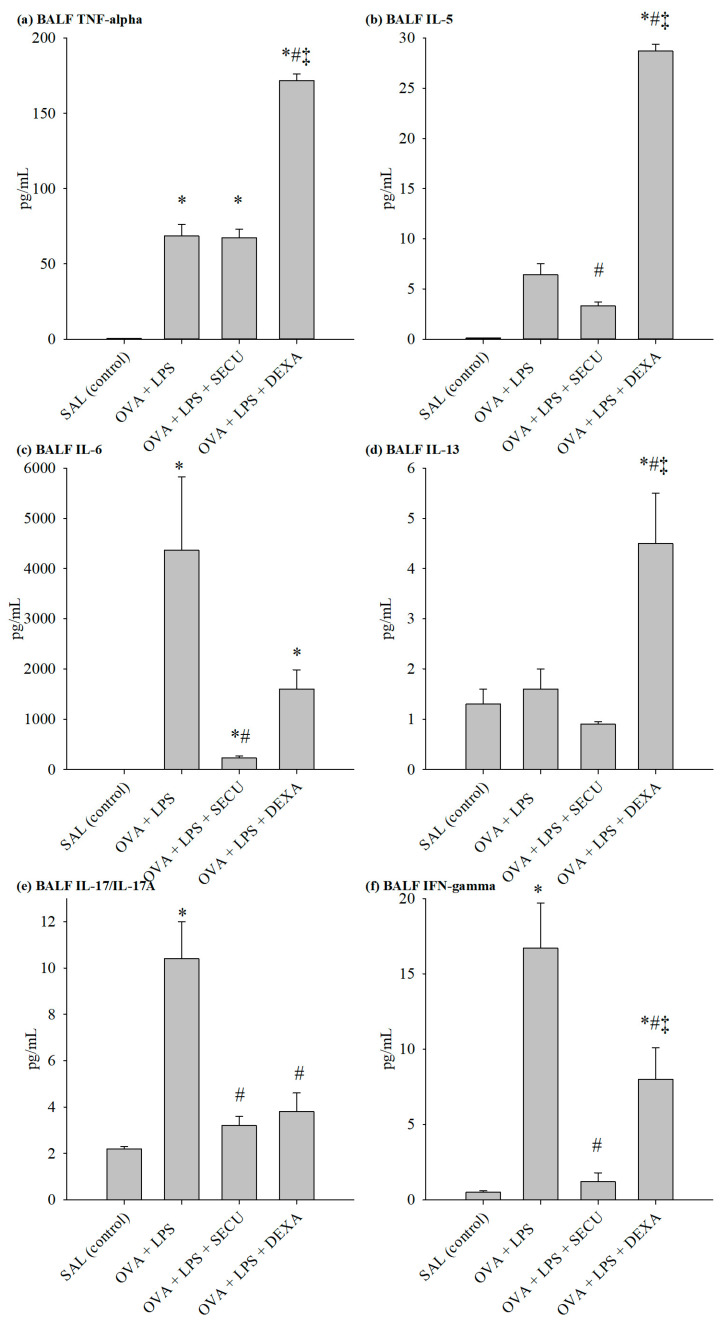
Cytokines concentration (pg/mL) in BALF: (**a**) TNF-α, (**b**) IL-5, (**c**) IL-6, (**d**) IL-13, (**e**) IL-17 and (**f**) IFN-γ. Values are represented as mean ± standard error; *n* = 6. One-way ANOVA: * *p* < 0.05 vs. SAL group; # *p* < 0.05 vs. OVA + LPS group; ‡ *p* < 0.05 vs. OVA + LPS + SECU group; followed by the Holm–Sidak method for multiple comparisons. SAL: normal control group; OVA + LPS: positive control for disease; OVA + LPS + SECU: Secukinumab treated group; OVA + LPS + DEXA: Dexamethasone treated group.

The evaluation of cytokines for specific Th1, Th2 and Th17 revealed an interesting interplay of these immune responses (Figure 4).

We also quantified the vascular endothelial growth factor (VEGF) and IL-4 in BALF (Table 1).

BALF determination of Th1 effector cytokines revealed the following: TNF-α level was not significantly influenced by SEC treatment, but the dexamethasone surprisingly increased its BALF concentration in comparison to the OVA + LPS group; on the other hand, SECU treatment decreased IFN-γ and IL-6 levels significantly, even more prominently than dexamethasone decreased the levels.

The same pattern for dexamethasone can be observed in the case of typical Th2 cytokines; it induces a marked increase in Il-4, Il-5 and Il-13 levels. As for SECU, it significantly inhibits the Il-5 increase in comparison to the OVA + LPS group. Evaluation of Il-17 levels for all four groups revealed a significant increase in the OVA + LPS group vs. the SAL group and a significant decrease under SECU or DEXA treatment. VEGF concentration suffered no significant modification under SECU treatment compared to the OVA-LPS group.

#### 2.2.3. Cytokines in Lung Tissue Homogenate (LTH)

**Figure 5:**
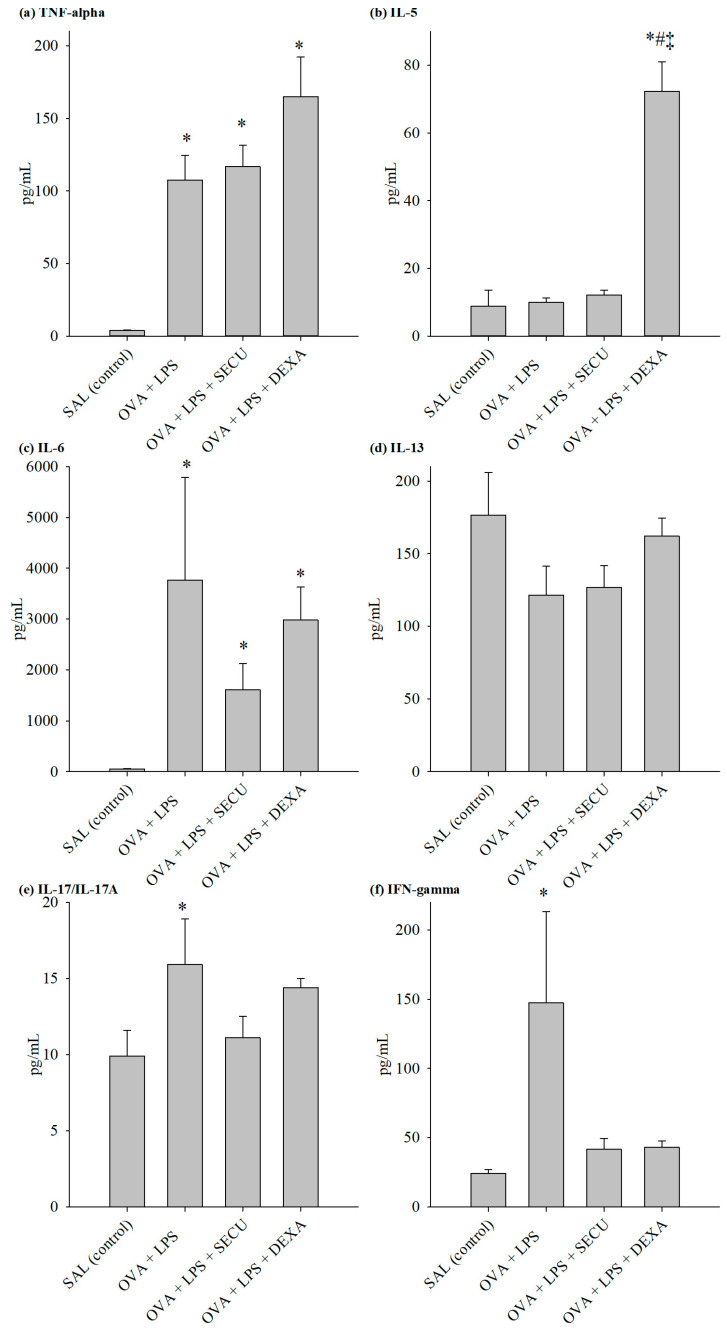
Cytokines concentration (pg/mL) in LTH: (**a**) TNF-α, (**b**) IL-5, (**c**) IL-6, (**d**) IL-13, (**e**) IL-17 and (**f**) IFN-γ. Values are represented as mean ± standard error; *n* = 6. One-way ANOVA: * *p* < 0.05 vs. SAL group; # *p* < 0.05 vs. OVA + LPS group; ‡ *p* < 0.05 vs. OVA + LPS + SECU group; followed by the Holm–Sidak method for multiple comparisons. SAL: normal control group; OVA + LPS: positive control for disease; OVA + LPS + SECU: Secukinumab treated group; OVA + LPS + DEXA: Dexamethasone treated group.

The evaluation of the cytokines for each of Th1, Th2 and Th17 revealed an interesting interplay of these immune responses (Figure 5). Data from cytokines assay in lung tissue homogenate were partly consistent with the results from BALF: TNF-α concentration was significantly increased in all OVA sensitized groups in comparison to the SAL group; as for Il-6, it was significantly decreased by the DEXA treatment only.

We also quantified the vascular endothelial growth factor (VEGF) and IL-4 in LTH (Table 2).

The marked increase in Il-4 and Il-5 levels induced by DEXA is also observed in lung tissue homogenate, excepting the insignificant value of Il-4 when compared with the OVA-LPS-SECU group. IFN-γ shows a significant decrease under SECU treatment.

Il-13 and Il-17 levels were not statistically significantly modified in OVA-LPS treated and untreated groups.

VEGF levels showed a significant decrease when compared with the SAL group.

#### 2.2.4. Histological Analysis

**Figure 6:**
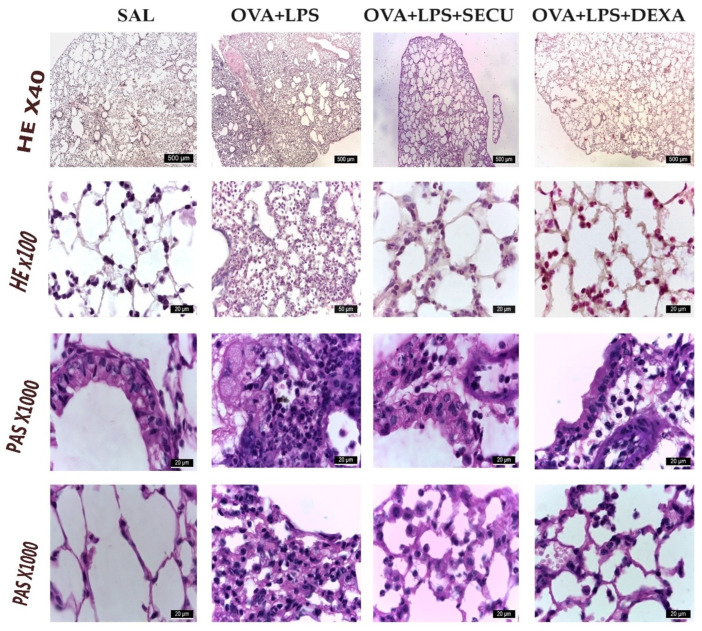
Representative HE and PAS-stained tissue sections of lungs and bronchia from mice in ovalbumin-induced asthma exacerbated with LPS administration. Original magnification for HE ×40 or ×100, for PAS: ×1000. SAL: normal control group; OVA + LPS: positive control for disease; OVA + LPS + SECU: Secukinumab treated group; OVA + LPS + DEXA: Dexamethasone treated group.

In the SAL group (Figure 6), normal lung structure is observed. Bronchi are lined by epithelium pseudostratified ciliated epithelium consisting of ciliated cells, club cells goblet cells and basal cells. In the lamina propria, there is a connective tissue. The height of the epithelium decreases as the epithelium expands and more branches of the bronchi reach the terminal bronchiola. In the vicinity of the bronchi, there are blood vessels and nerve threads surrounded by connective tissue. The pulmonary alveoli have a very thin wall formed of type I and type II alveolar cells. In the septal wall, there are alveolar macrophages.

The histological changes in the lung with OVA + LPS consist of an increase in the height and number of goblet cells of the pseudostratified ciliated epithelium lining the airways. The epithelium shows broken intercellular junctions, visible by light microscopy, perivascular and peribronchial oedema, infiltration with neutrophilic polymorphonuclear cells, eosinophils, lymphocytes and peribronchial macrophages. In the lumen of the bronchi, polymorphonuclear cells can be seen. Lung alveoli are collapsed, the septal wall is enlarged in diameter. Neutrophils, monocytes, eosinophils, alveolar macrophages and lymphocytes can be observed both in the septal wall and in the lumen of the alveoli.

In OVA + LPS + SECU, the number of neutrophils decreases significantly, remaining in low numbers around the bronchi. The lung alveoli have a thinner wall, a reduced number of figurative elements in the lumen or septal wall and collapsing of the lung alveoli rarely occurs.

OVA + LPS + DEXA shows a dense, smaller area compared to OVA + LPS + SECU, in the central area of the lobules, where the lung alveoli are collapsed and mostly infiltrated with neutrophils, lymphocytes and macrophages.

## 3. Discussion

Our experimental model of induced asthma with ALI overlap revealed a significantly increased level in TNF-α both in BALF and LTH. According to Amatya et al., Il-17 and TNF-α are acting in synergy during the inflammatory process [31]—this means that inhibition of Il-17 might indirectly decrease TNF-α pro-inflammatory stimulation and raises rightfully the interest for the potential of Il-17 targeting.

There are clear experimental results on Balb/c mice models from other authors in the larger field of either OVA-induced asthma or LPS-induced ALI. There are other experimental studies on BALF cytology and cytokine response (IL-4, Il-5, IL-13, IL-17 and IFN-γ) in animals only with individual exposure to OVA in OVA-induced asthma [29,32,33]; the impact of anti-Il-17 [33] or dexamethasone [29,32] treatment alone in this model was evaluated. Also for the LPS-induced ALI in Balb/c mice model, previous experimental studies of other authors on BALF cytology and cytokine response (IL-1β, IL-17 and TNF-α) evaluated individual exposure groups to LPS with or without anti-Il-17 treatment [5]. Consequently, to follow the goal of the 3Rs principles (Replacement, Reduction and Refinement), it was decided to restrain the number of experimental groups in this study design.

As acute potentially fatal asthma is an airway disease and ALI a pulmonary parenchymal disease, we attempted to evaluate the effects of SECU in both BALF and LTH in order to quantify better the impact on this particular pathology.

BALB/c mice are known to react sensitively when challenged with LPS [34]; this led, in our study, to a typical ALI inflammatory reaction with leukocyte invasion and upregulation of proinflammatory cytokines within hours to days consistent with the model proposed by Ehrentraut et al. [35]. The increased neutrophils percentage in the OVA + LPS, OVA + LPS + SECU and OVA + LPS + DEXA groups compared with the SAL group confirmed once more the ALI. However, probably the lower dose of SECU (10 mg/kg) and the study design were the reasons for a different outcome for SECU effects in the lung inflammation on severe sepsis model rats—the dose was double: 20 mg/kg [27]. The high concentration of neutrophils under DEXA may be caused by an induction of neutrophil recruitment into the airways documented for patients with mild asthma under oral but not inhaled route corticosteroid therapy [36] and by a potent inhibitor of neutrophil apoptosis that could be exerted by DEXA [37]. Our results are supported by the findings of Aubin Vega et al. on DEXA who failed to reduce the neutrophil infiltration in BALF [38].

The insignificant modification of macrophages under SECU in comparison to OVA-LPS can be considered a positive outcome given the fact that macrophages are also important players in the resolution of pulmonary inflammation and wound healing processes [39,40]. The lymphocytes and eosinophils were increased in all OVA sensitized groups confirming the proposed study model.

DEXA induced Il-4 increase may have multiple explanations: the above-mentioned increased neutrophils under DEXA corroborated with the ability of infections to induce IL-4 expression by neutrophils [41] and under DEXA treatment, increased eosinophils were able to release IL-4 containing granules [42]. Also increased Il-5 in the OVA-LPS-DEXA group might contribute through recruitment of an activation of eosinophils [43,44]. On the other hand, SECU revealed potential benefits by the inhibition of IL-5 at least in BALF.

Probably the lower dose of SECU (10 mg/kg) and the study design were the reasons for a different outcome in a recent study for SECU effects in the lung inflammation on severe sepsis model rats—the dose was double: 20 mg/kg [27].

Study limitations:

- there is a limited number of exposure groups in our study, and at least two other exposure groups, with LPS and OVA only, could have been helpful to understand better how the combined treatment deviates from the individual pathological condition; however, the study design was decided based on the already existing comparative results [1,5,45] for OVA and LPS and OVA + LPS; moreover, the goal of the 3Rs principles (Replacement, Reduction and Refinement) was followed;
- secondary to binding the LPS binding protein (LBP), LPS:LBP complexes ligate the TLR4 receptor located on the surface of many cell types, inducing a strong proinflammatory reaction with the upregulation of cytokine expression [46]; the results interpretation have to be completed with a certain degree of caution because mice share with humans only approximately 50% homology of the TLR4 receptor [34];
- overreliance on murine models can often be difficult to extrapolate to the complex and uniquely human disease of asthma; moreover, the use of female only animals may raise the question of the potential effect on male mice;
- this study lacks a deeper mechanistic exploration of how SECU modulates the immune response in the context of asthma and ALI, but the link between intra-/inter-cellular signaling and the level of cytokines/inflammatory markers is already approached in asthma [47,48,49,50] or in ALI [51] by other authors;
- Secukinumab is able to neutralize cynomolgus, rhesus and marmoset monkey IL-17A but not the rodent IL-17A.

Following exposure to LPS, a shift from Th2-derived airway eosinophilic inflammation to Th17 neutrophilic inflammation in an ovalbumin-sensitized murine asthma model is promoted. This modification in the inflammatory phenotype is strongly correlated with the increases in IL-17 and Th17 cells [52]. Dexamethasone (DEXA) showed protective effects against LPS-induced ALI related to the inhibition of NF-κB and indirect augmentation of IL-10 expression [30]. In the intravenous administration, the use of DEXA resulted in a statistically significant increase in the number of ventilator-free days among patients with COVID-19 and moderate or severe ARDS [53]. However, DEXA failed to reduce the neutrophil infiltration in BALF, mortality rates or lung edema [38].

Our results show similitudes with other findings regarding DEXA like the ability to promote and maintain Th17 differentiation in mice in vivo [54] and Th cells from patients with steroid-resistant asthma produce higher levels of IL-17 than from patients with steroid-sensitive asthma or healthy controls [55].

The impact of SECU and DEXA (as a positive control) by quantifying markers/cytokines for different types of immune responses (Th1, Th2, Th17) in BALF and in lung tissue homogenate (LTH) on a murine model of LPS-induced ALI overlapped on OVA sensitized allergic asthma. To avoid the potential bias in the qualitative/quantitative determination, we used the same Luminex Mouse Discovery Assay for both tests.

Tests (blood leukocyte panel, IgE levels, standard cytokine panel including Th1, Th2 and Th17 cytokines, together with allergen identification) that allow for the diagnosis of asthma endotype have to be carried out as routine care, with the findings documented in medical records. When common airborne infections infect patients with underlying allergic asthma, complex, diverse and context-dependent host-pathogen interactions take place [56].

Overall, the results are in line with the previous findings from the literature regarding the involvement of Il-17 in ALI pathogenesis.

The treatment guideline for asthma [57] offers multiple monoclonal antibodies-based treatment options for severe asthma in a phenotype-oriented manner, but there is a gap in the case of severe asthma characterized by the Th2-low/Th17 phenotype. Although anti-Il-17 administration in the later asthma phenotype [1,2,3,58] and in ALI [5] in murine models showed promising results, SECU effects, particularly, have not been tested yet. Moreover, in the clinical scenario of severe asthma exacerbated by infection/pneumonia that evolves to ALI, the targeting of Il-17 brings benefits not only in asthma, but may prevent or at least diminish the development of ALI.

Therapeutic measures are few and ineffective when infection has advanced to severe pneumonia and ALI because supportive care, such as oxygen therapy and mechanical ventilation, may worsen the condition. Severe disease is frequently caused by detrimental host responses to infection, and there are no host-response specific treatments that can reverse the condition. There is a need for novel strategies to combat co-infections between viruses and bacteria, particularly in light of the rise in bacterial strains resistant to antibiotics. These are worldwide issues for which finding sustainable solutions would also necessitate international cooperation. Beyond pathogen-specific targeted strategies, such as those being studied with bacteriophages, host-response regulation through precision medicines is still an alluring prospect for the future [59].

## 4. Materials and Methods

### 4.1. Chemicals and Antibodies

Chemicals and drugs used were Saline solution (NaCl 0.9%), Phosphate buffered saline solution (BioUltra), aluminum hydroxide (Sigma-Aldrich Chemie GmbH, Schnelldorf, Germany), ovalbumin (GRADE V, Sigma-Aldrich, St. Louis, MO, USA), NP-40 lysis buffer (Thermo Fisher Scientific, Lancashire, UK), LPS (Escherichia coli O127:B8, Sigma-Aldrich Chemie GmbH, Schnelldorf, Germany), Protease Inhibitor Cocktail (Promega, Madison, WI, USA), anti-IL-17 monoclonal antibody (Secukinumab, Cosentyx^®^, Novartis Pharma GmbH, Nuremberg, Germany), Escherichia coli 0127: B8 (Sigma-Aldrich, St. Louis, MO, USA), Ketamine Hydrochloride (Ketavet^®^ 100 mg/mL Solution for Injection, Zoetis UK Limited, London, UK) and xylazine hydrochloride (Sigma-Aldrich Chemie GmbH, Schnelldorf, Germany). Antibodies for sample processing were LEGEND MAX™ Mouse OVA Specific IgE ELISA Kit (BioLegend, San Diego, CA, USA) and Luminex Mouse Discovery Assay 8-Plex- IFNgamma, IL-4, IL-5, IL-6, IL-13, IL-17/IL17A, TNF-alpha, VEGF (R&D Systems, Minneapolis, MN, USA).

### 4.2. Animal Experimental Design

Study design and protocols were approved by the Research Ethics Committee of the “Grigore T. Popa” University of Medicine and Pharmacy of Iaşi, Romania (approval No. 332/17.09.2023).

A total of 24 female BALB/c mice (17–23 g) obtained from the “Cantacuzino” Institute Bucharest were handled in accordance with the Research Law No. 206/27.05.2004 (published by the National Council for Ethics of scientifical Research, Technological Development and Innovation 12.11.2020) and housed in the CEMEX facility of our university in sterile cages under regulated conditions of temperature (22 ± 3 °C), humidity (55 ± 5%) and 12 h/12 h day/night cycle. The mice were provided with adequate food and drinking water through the entire period of study. The decision to use only female mice for this study is sustained by the findings that female mice develop a more pronounced type of allergic airway inflammation than male mice after OVA challenge [60].

The animals were divided into four groups (*n* = 6 in each group):

- SAL group (normal control), which received inhalations with a sterile saline solution;
- OVA + LPS group (positive control for disease), which received inhalations of an ovalbumin solution and LPS instillation;
- OVA + LPS + SECU group, which received inhalations of an ovalbumin solution, LPS instillation and treatment with Secukinumab (10 mg/kg, subcutaneous);
- OVA + LPS + DEXA group (positive control for treatment), which received inhalations of an ovalbumin solution, LPS instillation and treatment with Dexamethasone (1 mg/kg, intraperitoneal).

#### 4.2.1. Sensitization Protocol

**Figure 7:**
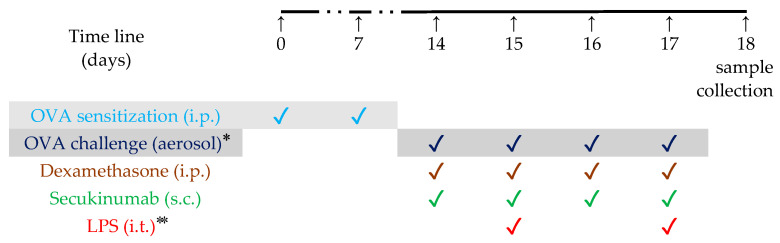
Chronological scheme of the experiments; * 1 h after Dexamethasone/Secukinumab administration, ** 1 h after OVA challenge.

The mice were acclimatized to in-house conditions for 7 days prior to the induction of allergic asthma/ALI and, subsequently, subjected to allergic asthma. OVA grade V was dissolved to a final concentration of 20 μg/mL in 500 μL sterile PBS per mouse and aluminum hydroxide (alum) was added to a concentration of 2 mg/mL, as described by Debeuf et al. [61]. The mixture was rotated for 30 min at room temperature on an end-over-end rotator. Mice from the OVA + LPS, OVA + LPS + SECU and OVA + LPS + DEXA groups received, via intraperitoneally injection, the OVA/alum solution in a total volume of 0.2 mL/per mouse on days 0 and 7 [62] and the SAL group received the same amount of a saline solution (NaCl 0.9%) and aluminum hydroxide (6 mg) also on days 0 and 7. On days 14, 15, 16 and 17, the animals were challenged for 25 min in an inhalation chamber with OVA by aerosolizing 10 mL of 1% OVA diluted in PBS (OVA + LPS, OVA + LPS + SECU and OVA + LPS + DEXA groups) or with saline solution (SAL group) as described by Debeuf et al. [61] (Figure 7).

#### 4.2.2. ALI Induction and Treatment

The intratracheal LPS administration on days 15 and 18 was performed at 1 h after de OVA aerosol and followed the protocol of Ehrentraut et al. [35] under general anesthesia with ketamine [80 mg/kg mouse bodyweight (BW)] and xylazine (10 mg/kg BW) intraperitoneally. On days 14, 15, 16 and 17, Secukinumab was administrated at a dose of 10 mg/kg subcutaneous 1 h prior to each OVA aerosol [26]. Dexamethasone was administrated at 1 mg/kg dose, via intraperitoneally injection, an hour prior to every OVA challenge and served as a positive control [63]. On day 18, all animals were euthanized in deep anesthesia (ketamine and xylazine overdose) by atlanto-occipital dislocation. The doses of SECU and DEXA were the same to those from similar experiments on mice [26,64].

#### 4.2.3. Blood Sampling for OVA-Specific IgE and BALF

When the pedal withdrawal reflex was lost (i.e., no response to a toe pinch), the mice were exsanguinated from the vena inguinalis. The blood was collected in 0.5 mL microcentrifuge tubes and the serum separated from the whole blood by centrifugation (10 min; 3000 revolutions per min; 4 °C) for determination of OVA-specific IgE using a Legend Max Mouse OVA Specific IgE ELISA kit (Biolegend, Sandiego, CA, USA), an Infinite 200 PRO M Plex Tecan plate reader (Tecan, Grodig, Austria) and Magellan v 7.4 software (Tecan, Grodig, Austria).

The trachea was cannulated and BALF was collected by three successive ice-cold PBS (0.5 mL) infusions-aspirations (total volume 1.5 mL) and then centrifuged at 250× *g* for 5 min at 4 °C. The resulting supernatant was transferred to another Eppendorf tube, centrifuged at 10,000× *g* for 15 min, aliquoted into tubes of 150 µL each and stored at −80 °C until the assessment of cytokines (RD-LXSAMSM-08 Luminex Mouse Discovery Assay 8-Plex: IFNγ, IL-4, IL-5, IL-6, IL-13, IL-17/IL17A, TNF-alpha, VEGF). From the residual cell pellet, after vortex mixing, 5 μL were used for slide preparation for differential counting and subsequently stained with May–Grünwald (Sigma-Aldrich, 1.01424) and Giemsa (Sigma-Aldrich, 1.09204). The mean cell count per microscopic field was determined using an optical microscope equipped with a dry objective lens (400× total magnification), through the examination of 10 fields. The differentiation of neutrophils, eosinophils, lymphocytes and macrophages was performed using an optical microscope, with an immersion objective lens (1000× total magnification), by assessing 200 cells.

#### 4.2.4. Lung Homogenate Analysis

The right lungs were harvested following the collection of lavage fluid and weighed, then transferred to 200 µL NP-40 lysis buffer (Thermo Fisher Scientific, Lancashire, UK) with Protease Inhibitor Cocktail (Promega, Madison, WI, USA) and kept on ice for 15 min. Manual grinding was performed using a 1 mL Wheaton Tenbroeck tissue grinder, on ice. The resultant homogenate was centrifuged at 10,000× *g* for 15 min, then 60 µL aliquots of the supernatant were stored at −80 °C until analyzed.

Cytokine quantification for IFN-γ, IL-4, IL-5, IL-6, IL-13, IL-17/IL17A, TNF-α and VEGF in lung homogenates was performed using a LXSAMSM-08 Luminex Mouse Premixed Multi-Analyte kit (R&D Systems, Minneapolis, MN, USA) and a Gen-Probe Luminex 100/200 xMAP platform (Austin, TX, USA). The samples were 1.25 × diluted in RD 6–52 and processed as indicated by the kit producer.

#### 4.2.5. Histological Analysis

After BALF collection, the left inferior lung lobe was removed and transferred to fixative 4% formaldehyde solution, dehydrated with ethyl alcohol of different concentrations, clarified with xylene, impregnated and paraffin-embedded, sectioned at 5 μm and stained with Hematoxylin Eosin (H.E.) and periodically with Schiff acid (PAS). Photomicrographs and measurement of histological structures were performed with a Leica microscope.

### 4.3. Statistical Analysis

The obtained data are presented as the means ± standard error (SE). Graphs are represented in bar formats with standard error and stars corresponding to significant differences compared to the SAL group. To determine the statistically significant differences between multiple groups, the parametric One-Way Analysis of Variance (ANOVA), followed by the Holm–Sidak method for multiple comparisons or the non-parametric Kruskal–Wallis One-Way Analysis of Variance on Ranks were used. All analyzes were performed using Statistical Package for the Social Sciences (SPSS) version 27.0 (SPSS Inc., Chicago, IL, USA) software. A *p*-value < 0.05 was considered statistically significant.

## 5. Conclusions

Our results suggest that in addition to dexamethasone, Secukinumab possesses the ability to modulate inflammatory cytokine release and to decrease Th17 response in ALI overlapped on exacerbated asthma in mice. Secukinumab long-term safety profile over five years was recently reviewed in other chronic diseases [56].

A better knowledge of host-pathogen interactions is desperately needed in order to find molecular biomarkers that might be utilized to guide precision medicine in a context- and time-specific way, given the variability of ARDS symptoms and the heterogeneity of the immune response. For each patient, invariant therapy options might not be the best option. Targeted research is therefore required to profile these patients for treatment plans that will impact and enhance individualized, effective healthcare with a lower medication burden during respiratory infections.

## Figures and Tables

**Table 1:** Absolute values of inflammatory markers in bronchoalveolar lavage fluid.

	SAL	OVA + LPS	OVA + LPS + SECU	OVA + LPS + DEXA
VEGF (pg/mL)	129.7 ± 12.8	354.7 ± 82.0	384.2 ± 47.0 *	390.3 ± 40.4 *
IL-4 (pg/mL)	6.1 ± 1.9	8.2 ± 1.8	9.6 ± 3.4	56.1 ± 5.2 *^#‡^

Values are represented as mean ± standard error; *n* = 6. * *p* < 0.05 vs. SAL group; # *p* < 0.05 vs. OVA + LPS group; ^‡^ *p* < 0.05 vs. OVA + LPS + SECU group; as determined by one-way ANOVA, followed by the Holm–Sidak method for multiple comparisons. SAL: normal control group; OVA + LPS: positive control for disease; OVA + LPS + SECU: Secukinumab treated group; OVA + LPS + DEXA: Dexamethasone treated group.

**Table 2:** Absolute values of inflammatory markers in lung tissue homogenate.

	SAL	OVA + LPS	OVA + LPS + SECU	OVA + LPS + DEXA
VEGF (pg/mL)	4155.9 ± 232.0	3134.1 ± 256.0 *	3811.2 ± 210.8	3696.4 ± 234.4
IL-4 (pg/mL)	102.3 ± 10.5	57.5 ± 8.3 *	76.9 ± 13.0	130.7 ± 11.0 ^#‡^

Values are represented as mean ± standard error; *n* = 6. * *p* < 0.05 vs. SAL group; # *p* < 0.05 vs. OVA + LPS group; ‡ *p* < 0.05 vs. OVA + LPS + SECU group; as determined by one-way ANOVA, followed by the Holm–Sidak method for multiple comparisons. SAL: normal control group; OVA + LPS: positive control for disease; OVA + LPS + SECU: Secukinumab treated group; OVA + LPS + DEXA: Dexamethasone treated group.

## Data Availability

The data presented in this study are available on request from the corresponding author.
